# A Tool to Calculate the Level of Occupancy in Indoor and Outdoor Spaces Using BLE and Open Data to Be Published in Real-Time

**DOI:** 10.3390/s20143916

**Published:** 2020-07-14

**Authors:** Montserrat Mateos Sánchez, Roberto Berjón Gallinas, M. Encarnación Beato Gutiérrez, Ana M. Fermoso García

**Affiliations:** Faculty of Computer Science, Universidad Pontificia de Salamanca, 37002 Salamanca, Spain; rberjonga@upsa.es (R.B.G.); ebeatogu@upsa.es (M.E.B.G.); afermosoga@upsa.es (A.M.F.G.)

**Keywords:** beacon, BLE, level of occupancy, open data, smartphone, localization, occupancy detection

## Abstract

The ability to know the precise level of occupancy in an indoor or outdoor space in real time could have multiple applications. It is a well-known problem for which a number of technologies have been proposed over time. The recent emergence of BLE beacon technology has provided a solution to the problem. This study presents a tool that uses beacons and user smartphones to determine the level of occupancy in indoor and outdoor spaces, providing real-time information that can be published as open data and subsequently used by other applications. The tool was tested in a university environment in real-life situations and has produced promising results in obtaining an occupancy count.

## 1. Introduction

The ability to know the precise level of occupancy of a section of a building in real time could have multiple applications, ranging from energy management within a building to emergency response plans. In the case of a university environment, it could be extremely useful to manage public spaces, such as classrooms, laboratories, library rooms or outdoor activities.

Indoor occupancy detection is a well-known problem that has been studied since the 1990s with the use of, for example, infrared sensors [1]. Since then, many other technologies have emerged, some of which have been applied to solve this very problem. Recent solutions have used RFID [2] or NFC [3] based technologies, although the most commonly used technology by far to solve this problem is WIFI [4,5]. RFID requires the installation of building antennas, as well as receiving devices, while NFC requires not only the placement of NFC labels throughout the building, but a high level of proximity to function properly, since it is a short range technology (<20 cm). The advantage of WIFI technology is that it does not require the addition of supplementary devices to the building, due to the current widespread use of this technology. The drawback, however, is a high level of energy consumption on the user’s device, usually a mobile phone, which is continuously scanning the WIFI. Moreover, the signal strength of a wireless device may also change over time, or an indoor environment may change. Recent studies are focusing on improving location accuracy of Wi-Fi fingerprinting localization from different approaches, with self-calibration time-reversal (TR) [6], using probabilistic [7] or Extreme Learning Machine (ELM) [8]. All of these technologies have certain drawbacks that can be solved, as explained below, with Bluetooth Low Energy (BLE).

Since the emergence of the new Bluetooth 4.0 Low Energy (BLE) [9], new applications of this technology appeared. This technology presents numerous advantages compared to previously used technologies. It is included in the majority of smartphones, has easy installation, low energy consumption, which requires only basic maintenance, and is low-cost, non-intrusive and has high performance. Since the emergence of this technology, several authors have already used Bluetooth beacons for indoor location or as part of different approaches to solve occupancy problems. For example, in [10,11,12,13] beacons were used to detect the presence of persons in a specific indoor region, although without detecting the precise number of individuals. Some authors [13] used a mobile app for their experiment, but it is only available for IOS devices. On the other hand, in [14,15] an exact number of people was detected by using a mobile application that receives the beacon signal, although all of these approaches are different to our solution. In this work, Conte et al. [14], pioneers in contributing solutions to count people based on BLE, do not use standard beacons but they customize iBeacon protocols using hardware based on Arduino, being difficult for use in real contexts. Moreover, [15] focuses its proposal in an absolutely different scenario, in which the solution is focused in the consumption of energy in buildings, so the problem of counting a precise number of individuals is not so important. In this work, the authors use Android devices and scene analysis for the detection of people. A more recent approach is the solution provided by [16], in which authors base their work on reducing the consumption of energy in an office, and where the workers delimited their working space, therefore making the localization easier. Besides, authors add energy meters in order to improve precision. Our study case is completely different to these approaches, since our work is focused on computing the level occupancy in laboratories, classrooms, study areas in the university and other outdoor spaces, with the goal of allowing students to know the level of occupancy of spaces in real-time so they can move to them if they are available. In addition, in our approach, the data of occupancy are published as open data for their use in other applications of the university, and also, these data can be used in applications to make data analysis, which will help decision making in order to plan timetables for opening hours. In other proposals, BLE beacons work with GPS for both indoor and outdoor detection [17].

Nevertheless, we have not come across any approach in the literature similar to our own where: (i) Bluetooth beacons are used to provide a high-accuracy count of individuals, whether indoors or outdoors; (ii) the system is low-cost because it uses only beacons and it is also non-intrusive because it is incorporated into the university app that both students and teachers should have installed; (iii) the occupancy information gathered for a specific region is accessible from any device with an internet connection; (iv) the information is published in real-time as open data for use in other applications, which we consider an essential factor for greater use.

The proposed solution assumes that most people have a smartphone, of which nearly 100% incorporate BLE 4.0 or later technology. Therefore, the use of Bluetooth beacons strategically placed in an outdoor or indoor area, together with the use of smartphones, makes it possible to detect presence and calculate the location of individuals, which then allows us to identify the level of occupancy in a specific area or space. The strategic placement of the beacons is calculated by triangulation algorithms [18]. The concept of trilateration is also used to determine whether a specific smartphone, and, by extension, a specific user, is located within the occupancy zone defined by the beacons.

The configuration data and beacon parameters, as well as the people counting information, must all be persistently stored. To this end, we have defined the data structures required to store the data and have implemented RESTful API [19] to access the data using a mobile phone.

Additionally, the occupancy data for every space are published in real time as open data that can later be used by another RESTful API. The set of open data to be published was defined and implemented following national and internationally established recommendations [20,21].

In order to verify the proposed solution, a prototype was implemented in a university to immediately identify the level of occupancy within a specific space, such as a classroom, laboratory or sports facility, among others. Two mobile applications were implemented for the prototype. The first allows for the configuration of beacons and, therefore, spaces. In other words, the beacons define the area where the people are counted to determine the level of occupancy. The second, which is installed in each user’s smartphone, determines whether a user is located within the established area, and then sends the data to the server in order to calculate the level of occupancy. This paper begins with an introduction of the BLE and beacon technology base used in the study. The algorithms used to implement the tool are then defined. This is followed by an explanation of the apps that were implemented to calculate the level of occupancy, as well as the set of open data defined within a university context for use by other applications. Finally, the test used in the study is presented along with the results obtained and the main conclusions.

## 2. Materials

### 2.1. BLE Technology and Beacon Devices

Beacons are a class of Bluetooth Low-Energy devices that broadcast Bluetooth signals. The Estimote [22] beacons used in this study have a strong ARM processor, powered by a button cell battery, which broadcasts radio signals through an integrated antenna. The signals broadcast by the beacons can be received by smartphones at close range and used to identify their location, as subsequently explained.

Given the characteristics of the beacons, they can be used to define a space or region when properly placed. Using trilateration algorithms from an individual’s mobile phone, it is possible to determine whether that person is located within the defined area and included among those counted.

Beacons provide the following parameters required to define the area in which the level of occupancy must be determined, and the number of individuals counted:

- UUID: A unique identifier for each beacon;
- Major: A parameter with a numerical value to identify and distinguish a group of beacons; all beacons from the same location (classroom, laboratory, area, etc.) will have the same value;
- Minor: A parameter with a numerical value to identify a single beacon within a group of beacons—identifies the beacon belonging to a group with a specific Major;
- Region: A set of beacons—regions can be defined according to the major and minor values. Once defined, all movements entering and exiting the region can be monitored (e.g., whether a device is within the range of the beacons defined by that region);
- Range: A parameter to define whether a device is within a specific range or distance. Range uses the concept of proximity estimation. Each beacon broadcasts a Bluetooth signal at a certain strength, which diminishes as the signal travels through the air. This allows the receiving device to estimate the approximate distance to the beacon. In fact, it is an inverse-square relationship, whereby if the distance of the beacon increases twofold, the intensity is reduced four times. As a result, the precision of the estimations decreases drastically as the distance increases. The range will, therefore, use the different intensities of the received signals to determine which beacons are likely to be closer and which are likely further from the device, and to classify the beacons into regions according to proximity—Immediate (strong signal within a few centimeters); Near (medium strength signal usually within a few meters); Far (weak signal, more than a few meters away).

### 2.2. Level Occupancy Tool. Algorithms

Calculating the level of occupancy in an area or region defined by beacons involves the following steps:

1. Establish and configure the area or region that will be monitored by beacons.
2. Determine the precise location of the user, and hence the smartphone, with regard to the beacons that make up the region or area.
3. Determine whether each user is located within or outside the previously defined and configured area.

#### 2.2.1. Establish and Configure the Region or Area to Be Monitored

**Figure 1:**
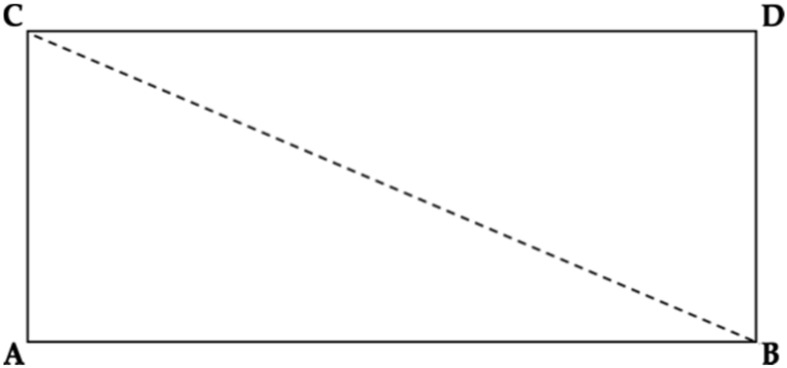
Square space formed by two triangles.

The area to be monitored will be configured using triangles theory [18]. The monitored area may be shaped as a square or rectangle and not necessarily a triangle, in which case two triangles will be used to cover the area, as shown in Figure 1. The same techniques will then be applied to both triangles.

**Figure 2:**
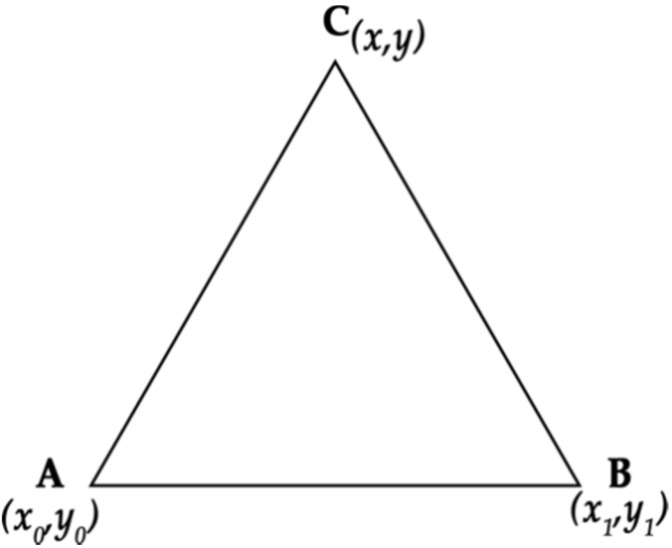
Triangle with a beacon located at each vertex.

A triangular space requires three beacons, each of which corresponds to the imaginary vertices of the triangle, as shown in Figure 2.

**Figure 3:**
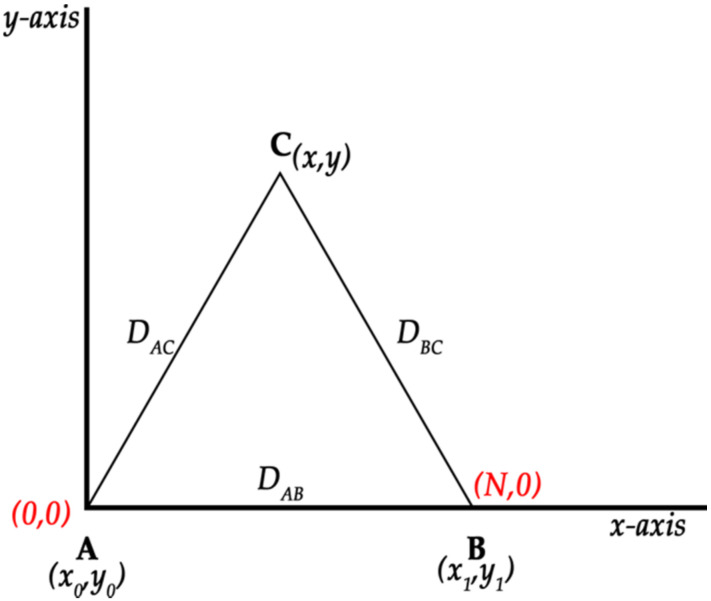
Calculation of the coordinates of the vertices of the triangle.

In order to calculate the vertices, we assume that the first beacon and the first vertex A are located at the imaginary coordinates (0, 0) and that vertex B is located on the *x*-axis. Since it is possible to determine the approximate distance between two beacons, when placed on the *x*-axis, the coordinates of point B are (N, 0) where N is the distance from beacon B to beacon A, as shown in Figure 3.

In order to calculate the coordinates of vertex C, we use the equation of circumference in a Cartesian plane whereby for each point P(x, y) of a circumference whose center is point Ce(a, b) and whose radius is r, the ordinary equation is:

(x − a)^2^ + (y − b)^2^ = r^2^ (1)

**Figure 4:**
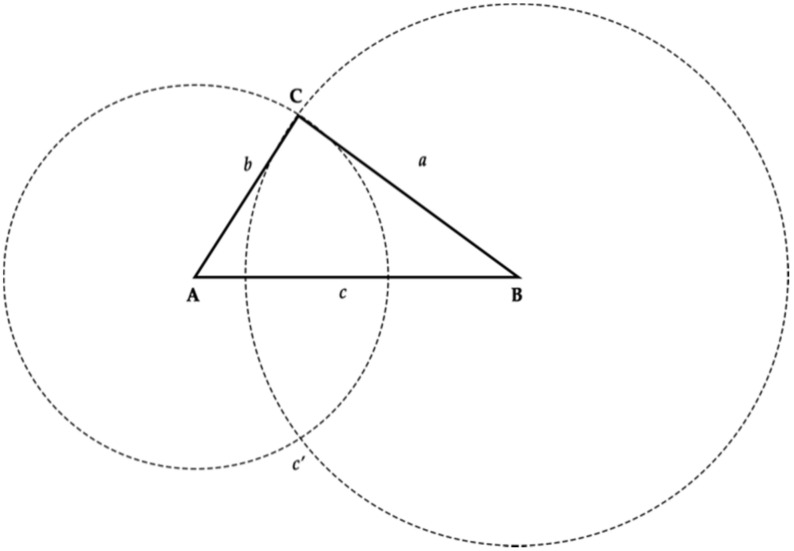
Calculate the third vertex of a triangle using two circumferences.

The intersection of the two circumferences, as shown in Figure 4, can then be used to identify the coordinates of the third vertex.

The vertices A (x_0_, y_0_) and B (x_1_, y_1_) are known and the distances D_AC_ and D_BC_ are provided by the beacons. This allows us to calculate the vertex C (x, y) using the following system of equations. This calculation defines the area or region whose level of occupancy we wish to know.

(x − x_0_)^2^ + (y − y_0_)^2^ = D_AC_^2^ (2)

(x − x_1_)^2^ + (y − y_1_)^2^ = D_BC_^2^ (3)

#### 2.2.2. Determine the Precise Location of a User with Respect to the Beacons That Make Up the Region

The next step is to determine the precise location of the user or smartphone with regard to the beacons that make up the defined region and establish whether the user is located within the defined space.

Once we have defined the region whose level of occupancy we wish to know, we can determine whether the user is located within or outside of the specific region by calculating both the coordinates of that user and the point coordinates with regard to the region made up of the beacons, after which we can confirm whether the user is within the defined occupancy zone.

**Figure 5:**
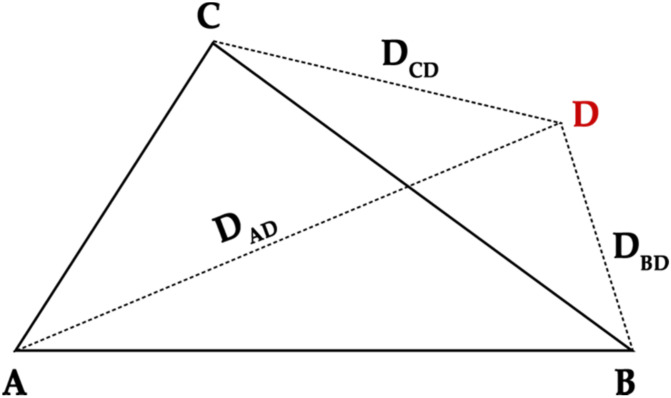
Calculation of the position of one point from the other three and the distance to each point.

Points A, B and C are the coordinates of the beacon that define the region, as calculated in the previous section. Point D is the receiver, meaning the user’s device or mobile phone, as shown in Figure 5.

In order to determine and calculate the point coordinates, the coordinates of points A, B and C must be known, as well as the distance from point D to each of the vertices of the triangle (A, B, C). This process can be carried out using trilateration [18], which performs an analogous calculation of the triangulation and uses the known locations of two or more points of reference, as well as the distance measured between the subject and each point of reference. When using only trilateration, at least three points of reference are required to determine the location relative to a point on a two-dimensional plane.

For this process to be carried out, the three known points, as previously explained, must be oriented so that one of the points represents the origin (0, 0), another point represents a point on the *x*-axis (N, 0), and finally, the third point (x_2_, y_2_) will be readjusted according to its position relative to the first two points.

Once points A, B and C have been calculated, it is possible to calculate one point relative to the other three as follows:

D_AD_^2^ = x^2^ + y^2^ (4)

D_BD_^2^ = (x − x_1_)^2^ + y^2^ (5)

D_BD_^2^ = (x − x_1_)^2^ + y^2^ (6)

The distances D_AD_, D_BD_ and D_CD_ are known because they are provided by the beacon. The previous equations can be used to calculate coordinates (x, y) for point D, which, in turn, allows us to identify the coordinates for the user or mobile terminal receiving the beacon signals.

#### 2.2.3. Determine Whether a User Is Located within or Outside of a Defined and Previously Configured Area

Once the coordinates for point D have been calculated to determine whether that point is found within or outside the area of the triangle with vertices A, B and C, we must then bear in mind that one point on a triangle can be described using the vertices of the triangle itself along with three coefficients—one for each vertex. Those three coefficients must fall within the interval [0, 1].

To this end, the coefficients α, β, and γ must be found within the interval [0, 1] so that the following equalities hold true:

D = α × A + β × B + γ × C(7)

α + β + γ = 1(8)

In other words, a system with three equations and three unknown variables—α, β, γ—is formed and is capable of being solved.

X = α × x_0_ + β × x_1_ + γ × x_2_ (9)

Y = α × y_0_ + β × y_1_ + γ × y_2_ (10)

α + β + γ = 1(11)

Note: We assume that point D is (x, y), point A is (x_0_, y_0_), point B is (x_1_, y_1_), and point C is (x_2_, y_2_).

Once this has been solved, if the values of α, β and γ are greater than 0, point D is located within the triangle. Otherwise, the point is located outside the monitored area.

## 3. Solution

### 3.1. Beacons and Algorithms

The proposed solution uses beacons and algorithms as was explain in previous section. All of the parameters of beacons were configured in the following way. The Major value is identical for all beacons within a specific area or space (classroom, laboratory, etc.) to be monitored. The Minor value will identify each beacon within an area and assign it with the same parameters that were calculated for it, such as its position. The Range can define the way the beacons are used with regard to their proximity. In order to test the result of each possible value, various tests were carried out. The Near value was eventually selected as the value to be implemented since the beacon responded as expected with this value. The Immediate value was discarded as it only responded when the device was very close to the beacon and did not allow for the possibility of defining a region, while the Far value was rather imprecise.

**Figure 6:**
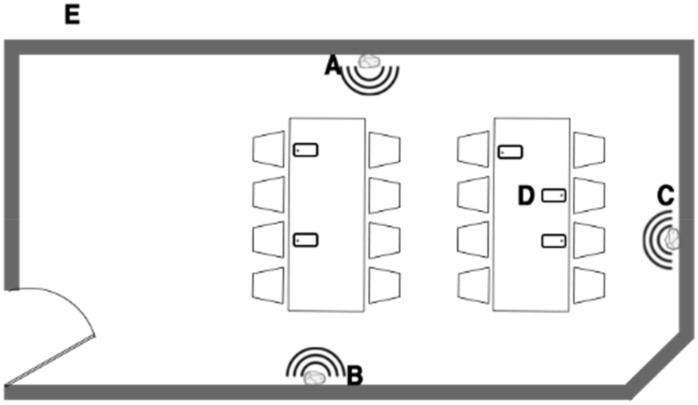
Beacon position in library study room.

In Figure 6, we can see how both algorithms are implemented in a test area or space—library study room—and also the position of each beacon (A, B and C). Point D (smartphone D) is inside the space defined by beacons A, B and C, and the system should detect it that it is inside, whereas point E is outside and the system should not detect it.

### 3.2. Data Management

The configuration parameters and operation of the beacons are persistently stored in a database so that they can be used at a later date by the app responsible for identifying the position of the device and determining whether a user is located within the occupancy zone.

Additionally, the data (date, time, identifier, etc.) generated by each user who enters or exits the defined area are persistently stored as well, with the aim of obtaining an occupancy count. Additionally, this information could be used to obtain insights into the use of spaces by means of statistical studies.

Web service requests are used to access and manage persistent data and information from a mobile terminal. An API based on RESTful [19] web services was used to access the data through Play! [23], an open source web application framework.

### 3.3. Open Data Set for a University Environment

As previously mentioned, all occupancy information is published as open data for future use and exploitation. The data were defined according to the principles for open data [20], in addition to recommendations by the Conference of Spanish Rectors (CRUE) in their commission of the ICT sector for the publication of open data in their document “Toward an open university” [21]. This document believes that classroom and laboratory equipment and their characteristics are likely to be published in open format. This set of data would fall within the framework of types of infrastructure and services.

This study aims to publish information in open data format about the laboratories, university classrooms and their basic characteristics, such as capacity, location, accessibility, and occupancy count at a particular moment.

The set of open data is specifically referred to as University Spaces, and contains the following data, as shown in Table 1 and Table 2.

This data set would complement and be related to the Characteristics Spaces data set, which contains the characteristics or elements for each space.

All data are accessible through an API based on RESTful web services for the open code Play! framework.

### 3.4. Mobile Applications

Finally, with regard to the necessary mobile applications, two additional applications were implemented for the Android and IOS platforms. The former can configure the space, while the second app is responsible for recording each visit into the space and can also determine the level of occupancy in that same space.

The app that configures the space includes an algorithm that has been defined (as detailed in Section 2.2) to delimit the region. This algorithm receives Bluetooth signals from the beacons placed within the area to be delineated, calculates the positions relative to the beacons, and sends configuration information to the server through requests placed to the defined web service. The beacons should be placed with a distance between them of about 7 or 8 m. The user-administrator of the area should setup the first beacon, approaching very close to the beacon (1 or 2 cm), after this, she should setup the next beacon in the same way but accept the configuration when the parameters (distance, signal power, etc.) provided by app are right, and so on with the rest of the beacons.

After, the smartphone of a user, when it gets into the area, receives this information in order to determine the location of the user and whether they are inside the defined space.

**Figure 7:**
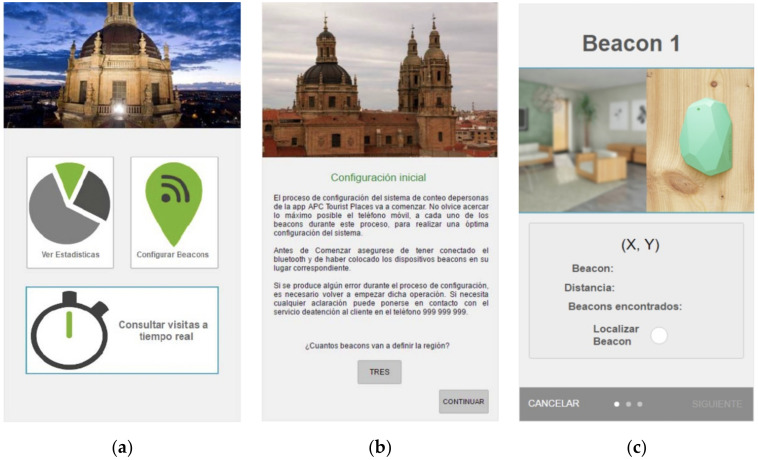
(**a**) Screen shot initial screen. (**b**) Screen shot initial configuration. (**c**) Screen shot beacon configuration.

The app, shown in Figure 7a, is only used when a new occupancy zone needs to be monitored and acts as a configuration assistant that selects the number of beacons that will be used to configure the area or space where an occupancy count is to be made, as shown in Figure 7b,c. The administrator will then approach each beacon, which the system configures automatically.

**Figure 8:**
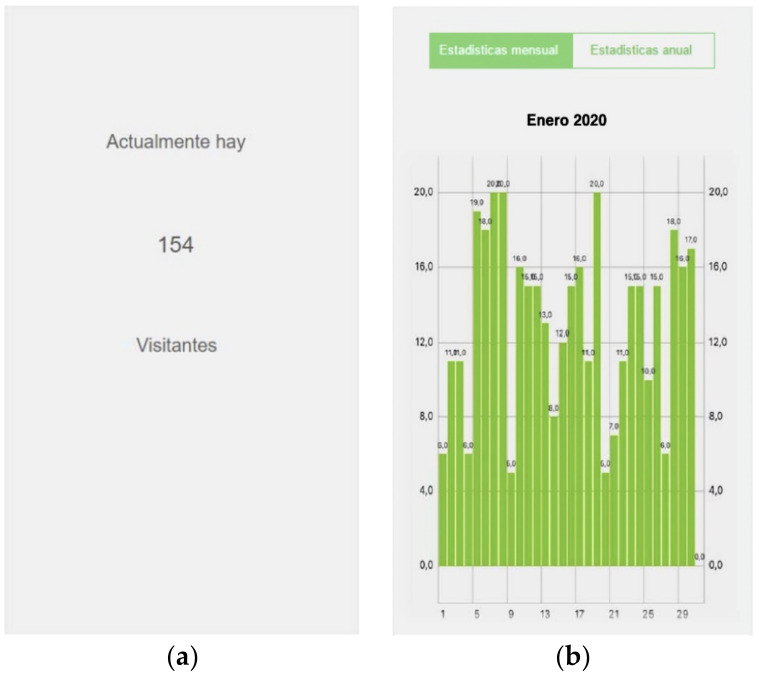
(**a**) Screen shot with the number of users in a defined space in real time. (**b**) Screen shot of the statistics on occupancy.

Additional functions were also included in the main functionality, including real time queries by users in the defined area, as shown in Figure 8a, and historical data consultations of occupancy, as shown in Figure 8b.

The second app, which will also receive the signals broadcast by the beacons, will use the operating data produced and configured with the previous app to implement the algorithms that will calculate the position of the mobile device according to the beacons, and determine whether it is located within the defined region. If the mobile device is located within the defined space, the information is sent to the server through a web service request.

## 4. Methods

The proposed system and the prototype implemented as a people counting tool to determine level of occupancy was used in both outdoor and indoor spaces. Various tests were carried out in different contexts and spaces in which there was interference from other signals—in indoor spaces, such as classrooms and library study rooms, laboratories (with interference from other signals) and in outdoor spaces, such as the University cloister and outdoor fitness tracks. The tests were carried out exactly in the following spaces:

- A squared-shaped laboratory classroom of about 40–50 square meters with 20 computers with WIFI interferences, tables, chairs and other office furniture. In this case, four beacons were placed. All beacons were located about 1.5 m from the floor. One of them was placed over a window ledge, and the rest on the wall.
- A square-shaped library study room of about 25 square meters, with office furniture and WIFI signal but without computers. Three beacons were located on the middle of the room walls.
- Additionally, the tool was tested in outdoor areas, such as a cloister with no interferences or furniture. Three beacons were placed in order to define a triangle-shaped area of about 30 square meters.
- Finally, it was tested in two outdoor fitness tracks, one of which is a stretching area without equipment and the other has fitness equipment—both with a size of about 50 square meters and, in both cases, four beacons were installed over metal posts specifically placed to locate beacons.

In all cases, the beacons were positioned in such way that they could be in the line of sight of smartphones because the results are much better than when the beacons were placed inside or behind some objects.

To measure the accurate of the system, a confusion matrix was used. The confusion matrix is based on values, such as true-positive, true-negative, false-positive and false-negative. It is used to calculate metrics that measure the accurate of classification approaches. Exactly, metrics used to test our solution are: accuracy; precision; recall; specificity; f-score.

Measurements were documented over a period of three weeks in each of the different defined spaces. Approximately 80–120 individuals entered these spaces daily. In order to calculate the false positives and true negatives, a controlled environment was created where 50 users were asked to closely approach each of the different defined areas.

In order to carry out the study, the identifications of the users (smartphone) were collected. The test was performed with different mobile devices with BLE 4.2 and 5.0. In total, 62.3% of users had an Android device and 37.7% had an IOS.

Every test and the results obtained every day during the three weeks were very similar. Although the tests were carried out during three weeks, in order to compute the values of the previously cited metrics, we have taken into consideration only the measurements taken the same day in which the tests under a controlled environment with 50 users were carried out. This decision was made with the goal that all of the values of the confusion matrix were taken in an environment under the same conditions (beacons battery, exactly the same location of beacons, same interferences, etc.).

The total number of users that entered in the spaces—in other words, users that were inside the areas in all spaces—was 117. The number of users that were not inside the areas but that were asked to closely approach each of the different defined spaces was 50. Table 3 provides greater detail about this. We can see how users were distributed by space and by operating system.

## 5. Results

The global results obtained are promising and we can affirm the system calculated the level of occupancy with high accuracy without significant differences in results between the Android and IOS devices.

In Table 4, we can see in a detailed way the findings obtained after calculating the values of metrics detailed in Section 4.

Observing the f-score metric values—the metric that provides a global accuracy and shows how robust and precise is the system—we can see that the values are between 0.935 and 0.965. These values are higher for the laboratory classroom and for the library study room than for the fitness track, so we can conclude the behavior of the system is slightly better for indoor than outdoor spaces in f-score terms.

If we take into consideration values obtained for accuracy—the global metric to know how correct the system is—we can also see in Table 4 a good behavior of the system with values over 0.96 in all cases. However, seeing values for precision (the real positive over the total positive that the system predicted), we can affirm the system detects those users that are inside spaces better, such as laboratory (0.983 with Android and 0.981 with IOS) and library study area (0.949 with Android and 0.952 with IOS) than those who are inside fitness tracks (0.904 with Android and 0.903 with IOS). So, we think that the system detects more positives improperly in outdoor than in indoor spaces, perhaps due to the fact that the area is not physically delimitated by walls.

About recall metric—the metric that shows how many users are correctly identified by the system of the total users that are in the space—it is shown that the system has a good behavior in all kind of spaces, being slightly better in outdoor spaces.

Finally, keeping in mind the specificity metric—the metric that measures the proportion of users identified correctly that had only been near but not actually inside—the system also has a good behavior in all spaces. Behavior is a little better in indoor spaces with interference from other signals, such as a computer room, than in outdoor spaces without interferences or walls, such as fitness track. Again, we think this could be due to the absence of walls, and so, the area could be diffusely delimited.

## 6. Discussion

This study presents a system and a completely functional tool to calculate the level of occupancy in outdoor and indoor spaces using BLE beacons. Algorithms were used to obtain an occupancy count of these spaces optimally and with a high level of accuracy. The cost of implementing this solution is very low, because beacon devices are cheap and only low cost beacons and mobiles of the users are necessary. In future works, some advanced techniques, such as fingerprinting, will be considered by the authors in order to get an exact accuracy of the level of occupancy for applications in which exact accuracy is required.

Additionally, the tool permits real-time queries on the level of occupancy of an area, providing these data in open format, along with other information of interest in a university environment. These data can be exploited in applications, providing them with added value. In this regard, many applications could benefit from using this set of data. For example, the university web application, which provides information about the opening and closing hours for specific areas (laboratory, classroom, study rooms), could also make information on the occupancy count prior to travelling to those areas available to users. Another possible application is to use historical data applied to machine learning techniques in order to obtain information on the busiest hours in each defined area, thus optimizing their use and adjusting schedules according to real-time demand.

It is also important to note that the user app used to determine the occupancy count can be integrated in the University app, providing an added value to the entire university community.

The system and tool designed for this study were implemented in a university environment, although its use can be extended to any other context in which it would be useful to know and exploit an occupancy count. Examples might include museum halls, malls, open air performances, etc.

## Figures and Tables

**Table 1:** University Spaces Data Set.

Data Name	Description
“Room Code”	Identifier code for classroom/laboratory
“Room Name”	Official classroom identifier according to signage nomenclature
“Campus Location”	Campus where the classroom is physically located
“Building Location”	Building on the campus where the classroom is physically located
Description	Classroom use and whether it includes student desks, computers, laboratory radio, etc.
Capacity	Total classroom capacity
Schedule	The opening hour is indicated, followed by a space and the closing hour. If the room opens/closes more than once a day, all opening and closing hours will be included, separated by a space
Accessibility	Whether accessible or not
“Real time Occupancy”	This information will be calculated by a counting tool and updates every 5 min

**Table 2:** Characteristics Spaces Data Set.

Data Name	Description
“Room code”	Identifier code for classroom/laboratory.
“Element identifier”	Identifier code for an element.
“Element description”	Description that clearly identifies element.
Quantity	Number of elements of a particular type in the classroom. This would be applicable only if able to be measured.
Availability	Possible values: Yes, No—as a function of whether the element is available.

**Table 3:** Detail about number of users participated in tests.

	Android		IOS	
	Number Users Entered	Number Users Closely, No Entered	Number Users Entered	Number Users Closely, No Entered
Laboratory classroom	39	31	23	19
Library study room	21	31	13	19
Fitness track	13	31	8	19

**Table 4:** Details of results for metrics of the tool proposed.

	Android			IOS		
	Laboratory Classroom	Library Study Room	Fitness Track	Laboratory Classroom	Library Study Room	Fitness Track
Accuracy	0.961	0.965	0.961	0.960	0.967	0.960
Precision	0.983	0.949	0.904	0.981	0.952	0.903
Recall	0.947	0.964	0.969	0.945	0.966	0.970
Specificity	0.979	0.965	0.957	0.978	0.967	0.956
F-Score	0.965	0.957	0.936	0.963	0.959	0.935
